# What is Behind the Rising Rates of Preterm Birth in the United States?

**DOI:** 10.5041/RMMJ.10065

**Published:** 2011-10-31

**Authors:** John D. Lantos, Diane S. Lauderdale

**Affiliations:** 1 Professor of Pediatrics, University of Missouri at Kansas City, and Director, Children’s Mercy Bioethics Center, Children’s Mercy Hospital, Kansas City, MO, USA; and; 2 Professor of Epidemiology, Department of Health Studies, University of Chicago, Chicago, IL, USA

**Keywords:** Preterm birth, prenatal care, infant mortality, epidemiology, bioethics

## Abstract

We review three decades of unsuccessful efforts by public policy-makers in the United States to develop programs to lower the rate of preterm birth. We analyze why these efforts had been unsuccessful. Finally, we will speculate about whether something has changed in the last few years that might finally bend the curve and reverse the trend of a steadily rising preterm birth rate.

## WHY ARE PRETERM BIRTH RATES SO HIGH IN THE UNITED STATES?

In 2007 and 2008, the rate of preterm birth in the United States dropped. This was the first time that the rate had dropped for 2 years in a row in over 30 years. The first drop was quite small—from 12.8% in 2006 to 12.7% in 2007—so public health officials were cautious about whether this was the start of a trend. But the second drop was larger—from 12.7% to 12.3%—and made it seem as if 30 years of efforts to lower the rate of preterm birth might finally be paying off. Still, questions remained. Why did preterm birth rates continue to rise so inexorably for so long? What led to the recent declines? Can the momentum be maintained, so that preterm birth rates continue to drop?

In order to examine what is behind these trends, we will review three decades of efforts by public policy-makers to develop programs to lower the rate of preterm birth. We will then examine some explanations for why these efforts had been so unsuccessful. Finally, we will speculate about whether something has changed in the last few years that might finally bend the curve and reverse the trend of a steadily rising preterm birth rate. We will talk only about preterm birth in the United States. Other industrialized countries have seen generally similar trends, for similar reasons, with some local variation. The situation in developing countries is very different.

First, we want to be clear about the terms we will be using, because many policies and outcome studies use different measures. In analysis of perinatal outcomes, the terms “preterm birth” and “low birth weight” are sometimes used almost interchangeably. Preterm birth is defined as birth before 37 weeks of gestation, measured from the first day of the pregnant woman’s last menstrual period. Low birth weight is defined as a weight less than 2,500 grams or about 5.5 pounds. Some babies are low-birth-weight without being preterm. Others are preterm but not low-birth-weight. Birth weight is easier to measure accurately than is gestational age. Thus, much data and many policies focus on low birth weight because accurate gestational ages are often not available.

## THE DISMAL HISTORY OF EFFORTS TO REDUCE PRETERM BIRTH RATES

For the 30 years prior to 2007, practitioners and policy-makers seemed powerless to reduce—or even stabilize—the rate of preterm birth in the United States. Between 1980 and 2006, the percentage of births that were preterm rose from 9.4% to 12.7%—a rise of nearly 30%.^1^ International comparisons further highlighted the failure of policy: the preterm rate in the United States is among the highest in the world and is similar to the rate in the least developed countries.^2^

The failure to reduce the rate of preterm birth over these years was not for lack of effort. Like a drum-beat, national commissions periodically recognized and highlighted preterm birth as a significant medical and public health problem. Their reports invariably set ambitious goals of reducing preterm birth or low birth weight in the foreseeable future and recommended concrete mechanisms for achieving those goals. One of the first of such reports, from the Institute of Medicine (IOM) in 1985, was entitled “Preventing Low Birth Weight.” That report laid out the stark facts:

Low birth weight is a major determinant of infant mortality in the United States … In addition to increasing the risk of mortality, low birth weight also increases the risk of illness … The association of neurodevelopmental handicaps and congenital anomalies with low birth weight has been well established; low birth weight infants also may be susceptible to a wide range of other conditions, such as lower respiratory tract infections, learning disorders, behavior problems, and complications of neonatal intensive care interventions.^3^

The report argued that better access to prenatal care would lower the rate of low birth weight and preterm birth, and that programs to improve access to prenatal care would be cost-saving. It argued for the following causal chain: 1) poor people who did not have health insurance were without access to prenatal care; 2) programs to provide insurance to poor people would lead to higher rates of prenatal care; 3) higher rates of prenatal care would lead to lower rates of preterm birth; 4) lower rates of preterm birth would mean less need for expensive neonatal intensive care, which was already paid for by government insurance programs; therefore, 5) increased access to prenatal care would lead to a net cost saving for government insurance programs. The IOM estimated that every dollar spent on prenatal care would save $3.37 in neonatal care expenses. This led one legislator to conclude: “It is not often that a person in public life gets to say, ‘I know how to save the lives of American children and save taxpayer money at the same time’.”^4^

In response to this report, the United States Congress passed legislation in the late 1980s that provided funding to expand the Medicaid program—a government health insurance program for the poor—in order to increase the number of poor women eligible for free access to prenatal care. This legislation had bipartisan congressional support and was signed into law by Republican President George W. Bush.

In one sense, these Medicaid expansions worked. More women did, in fact, enroll in Medicaid, and more of these women received prenatal care. From 1990 to 2003, the percentage of women who enrolled in prenatal care during the first trimester of pregnancy increased. The increases were largest in the highest-risk groups—7% for non-Hispanic white women, 24% for non-Hispanic black women, and 29% for Hispanic women. The percentage of pregnant women who did not receive any prenatal care was cut in half.^5^

In another sense, however, the policies seemed to be a dismal failure. National rates of both preterm birth and low-birth-weight birth continued to rise.

In 1991, the Surgeon-General of the United States issued a report, *Healthy People 2000*, setting 10-year goals for the nation’s health. One of the goals was to reduce the rate of low-birth-weight births from 6.9% to 5%. Over the ensuing decade, the rate rose from 6.9% to 7.6%.^6^ Undaunted, the Surgeon-General issued a new set of goals, *Healthy People 2010*, calling once again for a goal reducing low birth weight to 5%. In addition, this report called for a reduction in preterm birth from 11.6% to 7.6%. Over the next years, both low birth weight and preterm birth continued to rise.

In 2007, the IOM issued a follow-up to its 1985 report. Once again, they presented the compelling case for a new national effort to reduce the rate of preterm birth. They noted:

Infants born preterm are at greater risk than infants born at term for mortality and a variety of health and developmental problems. Complications include acute respiratory, gastrointestinal, immunologic, central nervous system, hearing, and vision problems, as well as longer-term motor, cognitive, visual, hearing, behavioral, social-emotional, health, and growth problems. The birth of a preterm infant can also bring considerable emotional and economic costs to families and have implications for public-sector services, such as health insurance, educational, and other social support systems. The annual societal economic burden associated with preterm birth in the United States was at least $26.2 billion in 2005.^7^

The next year, the Surgeon-General of the United States echoed the IOM’s call for more attention and research on the problem of preterm birth.^8^ And the preterm birth rate continued to rise.

Over all these years, and in most of these reports, the fundamental analysis and understanding of the basic nature of the problem of preterm birth and the consequences of such births have remained largely unchanged. Prenatal care was seen as a fundamentally *preventive* intervention. It was assumed that, if women got timely and comprehensive prenatal care, they would be less likely to deliver a baby too soon or deliver one that was too small. This belief persisted, even as evidence accumulated that improved access to and utilization of prenatal care did not reduce the rate of preterm or low-birth-weight births. As a preventive measure, prenatal care was clearly not working the way it was supposed to work.

Broadly speaking, there are three sorts of explanations for the trends in preterm birth rates. One explanation is that the interventions that we have used to try to lower the rate of preterm birth—primarily, the interventions that collectively are known as prenatal care—simply do not work. By this view, prenatal care itself needs to be redesigned to include only evidence-based interventions. Some recent reviews carefully evaluate various components of prenatal care in order to determine what actually works.^9^ They suggest ways to redesign prenatal care to make it more effective.

Another explanation for rising rates of preterm birth is that changes in the demographics of childbearing in the United States (and most of the developed world) have led to more high-risk pregnancies than ever before. Specifically, more women are delaying childbearing until they are in their 30s or 40s. Older women are known to have higher rates of both infertility^10^ and preterm birth.^11^ Treatment of infertility is associated with higher rates of multiple pregnancies, which are also associated with higher rates of preterm birth. So, by this view, we have more preterm births because we have more delayed pregnancy, infertility, and multiple pregnancies.

A third, related, explanation for the rise in preterm birth rates is that the rise is driven by medically induced preterm births—either by C-section or by pharmacologic induction of labor. This, then, leads to debates about whether such medical inductions are necessary or beneficial. Many critics of modern obstetrics see these medically induced preterm births as unnecessary and harmful. Others think that many, perhaps most, medically induced preterm births are beneficial.

We offer a fourth explanation, one that is related to and intertwined with the others. It may be that both the process and the outcomes of prenatal care have been misunderstood. It may be working, but working in a different way than it was once thought to work. Often, today, prenatal care allows the diagnosis of fetal problems or of maternal conditions that put the fetus at risk. Such diagnoses may lead to a medically induced preterm birth. When done appropriately, medically induced preterm births can lower the rate of both stillbirth and neonatal morbidity and mortality.^12^ Thus, better prenatal care might actually cause more preterm birth, but the increase in preterm birth might lead to decreased rates of both fetal and infant mortality. By this view, prenatal care should be seen less as a *preventive* treatment and more *an intervention* designed to identify and respond to problems that threaten the health of fetuses.

We will discuss each of these explanations and show how they might each be a part of the story. Finally, we analyze the implications of these analyses.

## DOES PRENATAL CARE WORK?

In the 1980s, the conventional wisdom was that better access to prenatal care would lead to lower rates of preterm birth and lower costs. The studies that led to this conventional wisdom generally compared women who received little or no prenatal care with women who received adequate prenatal care. In those studies, the women who received adequate prenatal care had dramatically better outcomes. For example, Leveno and colleagues published such an analysis in 1985: “Women seeking prenatal care had a significantly decreased incidence of low birth weight infants compared with those without such care … Prenatal care was associated with a 50% decrease in costs for each infant.”^13^

In a 1986 study, Moore and colleagues studied infants who were born at the University of California at San Diego. They compared infants whose mothers had received fewer than three prenatal visits with those whose mothers had received care in a comprehensive perinatal program. They showed:

When the total inpatient hospital charges were tabulated for each mother-baby pair, the cost of perinatal care for the group receiving no care ($5168 per pair) was significantly higher than the cost for patients in the Comprehensive Perinatal Program ($2974 per pair, *P*<0.001) including an antenatal charge of $600 in the Comprehensive Perinatal Program. The excess cost for delivery of 400 women receiving no care per year in the study hospital was $877,600.^14^

Joyce and colleagues, in a study done for the National Bureau of Economic Research, compared prenatal care with other interventions that might also reduce infant mortality. They compared teenage family planning, the supplemental food program for women, infants, and children (WIC), the use of community health centers and maternal and infant care projects, abortion, prenatal care, and neonatal intensive care. Their primary outcome measure was dollars (1984 dollars) per life saved. They showed that prenatal care was the most cost-effective of all these interventions, with a cost of about $30,000 per life saved. By contrast, neonatal intensive care, by their estimates, cost over $2 million per life saved.^15^

These studies defined the conventional wisdom. But the policies that they engendered did not lead to the expected outcomes.

Dubay and colleagues analyzed the effect of these expansions in the Medicaid program on access to prenatal care and birth outcomes. After the expansions, more women enrolled in early and comprehensive prenatal care. But there was no decrease in the rate of low birth weight. The researchers concluded: “The emerging lesson from the Medicaid expansions, however, is that increased access to primary care is not adequate if the goal is to narrow the gap in newborn health between poor and non-poor populations.”^16^

Ray and colleagues studied the effect of Medicaid expansions in Tennessee. They concluded: “In Tennessee, the Medicaid expansions materially increased enrollment and use of prenatal care among high-risk women, but did not reduce the likelihood of preterm birth.”^17^ Kaestner, in an analysis of national data, found little effect of the Medicaid expansions on birth outcomes and questioned the efficacy of these expansions.^18^

An insightful 1994 essay by Huntington and Connell suggested why. They pointed out that most of the earlier studies showing that prenatal care would be efficacious and cost-saving had serious methodologic flaws.^19^ In particular, they were confounded by selection bias which led to speculative estimates of the effectiveness of prenatal care in reducing low birth weight for women who would not typically have sought prenatal care. This led to underestimates of the true cost, and the true effectiveness, of comprehensive prenatal care for the highest-risk women, and an oversimplification of the relationship between prenatal care utilization, birth outcomes, and actual cost savings. As a result, they conclude: “The current public perception of prenatal care oversimplifies the difficulties of delivering prenatal care to women who do not now receive it, overestimates the benefits of prenatal care, and contributes to the medicalization of complex social problems.”

In response, researchers and practitioners developed and tested new and innovative ways to deliver more comprehensive prenatal care to the highest-risk women. They carried out randomized trials of different combinations of prenatal interventions. The programs included better social support, consultation with expert nutritionists, smoking cessation programs, stress reduction, subsidized transportation to clinic, comprehensive screening for vaginal and cervical infections, and other interventions. The goal of these studies was to come up with the absolute ideal of comprehensive prenatal care for the women at highest risk for bad outcomes. In short, they tried to both define a new approach to prenatal care and propose that it become the standard of care.

Eight such trials were summarized by Stevens-Simon in a 1999 meta-analysis. Overall, the trials enrolled nearly 10,000 pregnant women. Women who were offered psychological counseling, nutritional counseling, transportation, social support, comprehensive medical screening, and state-of-the-art obstetric services were compared with women who were offered “standard” prenatal care. Stevens-Simon concluded:

Although observational and quasi-experimental studies have produced a large volume of circumstantial evidence supporting the notion that comprehensive, multicomponent prenatal care prevents low birth weight, studies employing rigorous investigative methods have consistently failed to confirm the efficacy of this intervention strategy.^20^

Lu and colleagues did a similar analysis.^21^ They “reviewed original research, systematic reviews, meta-analyses and commentaries for evidence of effectiveness of the three core components of prenatal care—risk assessment, health promotion and medical and psychosocial interventions—for preventing the two constituents of LBW: preterm birth and intrauterine growth restriction (IUGR)”. They concluded that only two components of prenatal care—smoking cessation programs and antenatal corticosteroid therapy—reduced the rate of preterm delivery. Many other interventions, including bed rest, hydration, sedation, cerclage, progesterone supplementation, antibiotic treatment, psycho-social support, tocolysis, and home visitation, had insufficient evidence to show efficacy. They pessimistically concluded: “Neither preterm birth nor intrauterine growth retardation can be effectively prevented by prenatal care in its present form. Preventing LBW will require reconceptualization of prenatal care as part of a longitudinally and contextually integrated strategy to promote optimal development of women’s reproductive health not only during pregnancy, but over the life course.”

Perhaps one of the reasons why prenatal care did not work as well as it had been predicted to work was because pregnancy itself was changing. In particular, there is some evidence that the changing demographics of childbirth have led to more and more high-risk pregnancies.

## THE CHANGING DEMOGRAPHICS OF CHILDBEARING

Over the last three decades, as prenatal care programs were expanding and preterm birth rates were rising, the demographics of childbearing were also changing. Two particular changes were noted. First, the widespread availability of safe and effective contraception—along with social changes—led many women to delay childbearing into their 30s or 40s. Second, more and more women with infertility problems were using ovarian stimulatory drugs or *in-vitro* fertilization. Both older maternal age and assisted reproduction are associated with higher rates of preterm birth.

Since 1970, the fertility rate has gone steadily down for women under 30 and steadily up for women over 30. The average age at childbearing has been rising for the last 50 years. The mean age of women at the time of their *first pregnancy* increased by nearly 4 years between 1968 and 2002, from 21.4 to 25.1 years of age. The mean age at childbearing for *all pregnancies* rose over those years from 24.9 to 27.3. Over those years, the percentage of first births to women over 35 years of age went from less than 1% to 4%.^22^

Delaying childbearing is associated with higher rates of infertility.^23^ Infertility leads to the use of ovarian stimulation drugs and *in-vitro* fertilization.^24^ These treatments, in turn, are associated with higher rates of multiple pregnancies and higher rates of preterm birth. Although infants conceived with assisted reproductive technology (ART) accounted for only about 1% of the total births in the United States in 2003, the proportion of twins and triplets or higher order multiples attributed to ART were 16% and 44%, respectively.^25^ As fertility clinics limit the number of embryos transferred, rates of multiple pregnancies associated with *in-vitro* fertilization have leveled off.^26^

But these are not the only changes in the demographics of childbearing over these years. There has been a large increase in the percentage of births to unmarried women. This trend has been apparent since at least 1940, when only about 5% of births in the United States were to unmarried women. In 2007, 40% of births were to unmarried women.^27^ The rise in births to unmarried women is not a result of a rise in teen pregnancy, since those rates have been falling slowly but steadily over the same time period.^28^ In 1970, nearly 40% of first births were to mothers under the age of 20. In 2006, only 21% of first births were to teens.

In 2007, fertility rates were highest for Hispanic women (102/1,000) and lowest for non-Hispanic white women (60/1,000).^29^ The number of Hispanics in the population has risen steadily over the last 30 years, from 4.7% in 1970 to 15.5% in 2010. The high fertility rate among Hispanics thus contributes disproportionately to the overall birth rate.

We analyzed the effects of these changing demographics on overall rates of preterm birth, and showed that, taken together, these shifts in demographics cancel each other out as explanations for rising preterm birth.^30^ Using linked birth and death certificates, we showed that, if the demographic make-up of the childbearing population in the United States had not changed since 1980, the rates of preterm birth would likely have been the same. Instead, we suggest, the rising rates of preterm birth are accounted for by changes in obstetrics over these years.

## CHANGES IN OBSTETRICS

Obstetrics is clearly changing. Perhaps the most easily measurable indicator of the changes in obstetrics over the last 40years is the rate of C-sections. In 1970 in the United States, 5.1% of deliveries were by C-section. By 1980, C-sections were performed in 16% of all deliveries. The rate leveled off between 15% and 22% in the 1980s and then began to rise dramatically once again. By 2006, it reached 31% of deliveries.

Many C-sections are done preterm, and the rate of C-sections in preterm births has been rising along with the rate in term births. In 1991, 25% of singleton preterm births were by C-section. By 2006, the percentage had risen to 36.9%. Over these years, C-section rates rose in all age groups, in all racial groups, and among women with all different types of health insurance, including no insurance. C-section rates rose as fast among women with no identifiable risk factors as among high-risk women (though the overall rate among low-risk women is much lower).^31^ Clearly, the rise in obstetrical interventions is one of the reasons why preterm birth rates are rising. MacDorman and colleagues showed that, in 2006, nearly half of very preterm deliveries and about one-third of late preterm deliveries were by C-section. Another 15% of preterm deliveries followed medical induction of labor.^32^

Is this necessarily a bad thing? The answer is not so clear. Some argue that medically induced preterm deliveries are preventing intrauterine fetal deaths, particularly fetal deaths in the third trimester of pregnancy. The data to support such claims come from epidemiologic studies of associations between medically induced preterm birth and fetal death rates.

Over the last few decades, fetal death rates have fallen dramatically in the United States. In 1985, the fetal death rate was 7.8/1,000 pregnancies. By 2004, it had declined to 6.2/1,000, a 20% drop. The drop in late fetal deaths, those after 27 weeks of gestation, was even more dramatic. Rates fell from 4.95/1,000 to 3.1/1,000, a 37% drop.^33^ Two recent reports analyze the association between rising rates of C-sections and falling perinatal mortality rates. Ananth and Vintzileos show that a rise in preterm C-section rates from 1990 through 2004 was associated with a reduction in stillbirths by 5.8%, 14.2%, and 23.1% at 24–27, 28–33, and 34–36 weeks, respectively.^34^

Fetal mortality rates (after 20 weeks of gestation) and neonatal mortality rates (up to 28 days of age) can be combined into a “perinatal mortality rate.” That has fallen from 14.6/1,000 live 20-week fetuses in 1985 to 10.7/1,000 in 2004, a 27% drop.

What accounts for this decline in fetal mortality, which is greatest after 28 weeks of gestation? According to a recent analysis by the Centers for Disease Control, much of the decline can be attributed to improved access to prenatal care leading to better fetal screening and the early diagnosis of pregnancy problems. The report highlights “fetal imaging, prevention of perinatal infections, effective treatment of maternal medical conditions such as diabetes and chronic hypertension, and more aggressive management of labor and delivery” as likely contributors to improved fetal survival.^35^

Such an analysis might explain, in part, the relationship between improved access to prenatal care, decreased rates of fetal demise, and increased rates of preterm birth. For women in high-risk groups—categorized either demographically or medically—more intensive prenatal care with more frequent screening of both the pregnant woman and the fetus may lead to earlier diagnosis of medical risk factors or fetal distress. This, in turn, might lead to a clinical decision to induce delivery.

Joseph has used such data to propose a new theory of obstetrics. He argues the efficacy of obstetrics should be assessed by evaluating outcomes for all fetuses at risk—that is, all fetuses at 20 weeks of gestation or above.^36^ By this approach, the proper measure of the success of obstetrics should not be preterm birth rates or infant mortality. Instead, it should assess survival rates for all living fetuses from 20 weeks of gestation onward. He explains the difference as follows:

Under the traditional model of perinatal death, neonatal deaths occur among infants in the first month after birth and the unborn fetus is not a candidate for neonatal death. However from a broad biological, obstetric and ultimately epidemiologic point of view, a fetus at any gestation is at risk of stillbirth and neonatal death at that gestation. If one considers a woman at 28 weeks gestation with severe preeclampsia and fetal compromise, the risk of stillbirth is easy to conceptualize. The risk of neonatal death is substantial as well and can follow either premature labor or medically indicated delivery. The same risks apply in concept to a woman with a healthy pregnancy at 28 weeks gestation, despite the magnitude of the risks being considerably smaller. Thus, although neonatal deaths literally occur among infants, fetuses can be considered candidates for neonatal death as well.^36^

Lisonkova and colleagues analyzed pregnancy outcome data in the United States and Canada, using this approach, and showed that higher rates of medically induced preterm births were associated with decreased fetal mortality, infant mortality, and severe neonatal morbidity.^37^

Such data suggest that the rise in preterm birth may not be such a bad thing. It may reflect better obstetrical care with more sensitive assessments of fetal distress. When coupled with excellent neonatal intensive care, it may lead to improved outcomes for babies compared to an approach to obstetrics that is oriented towards maximizing rates of term birth.

Some have raised concerns, however, about the over-use of medically induced preterm birth, the consequent rise in near-term deliveries, and the morbidity associated with near-term birth, even when excellent neonatal care is available. For example, Woythaler and colleagues studied neurodevelopmental outcomes at 2 years for babies born after 37 weeks and those born between 34 and 37 weeks (“late preterm”). They showed that the late preterm babies had more physical, cognitive, and developmental delay.^38^ Of course, such studies have the same problems of confounding as do all non-randomized trials. We do not know if outcomes were worse because the babies were born preterm, or whether the babies were born preterm because they had problems that led to poorer neurodevelopmental outcomes.

## PRENATAL CARE, PRETERM BIRTH, INFANT MORTALITY, AND FETAL MORTALITY

Overall, this review suggests a complex relationship between changing demographics, changes in prenatal care and obstetrics, fetal mortality and infant mortality. Some of the changes in the demographics of childbearing—particularly delayed childbearing and increased average maternal age—clearly lead to more high-risk pregnancies. Other changes, such as increases in the education levels of pregnant women, lead to fewer high-risk pregnancies. Changes in obstetrics that make the management of high-risk pregnancies better inevitably spill over into obstetric practice generally. These changes make it possible to monitor the fetus more closely and to diagnose more fetal problems. It is hard to know which babies will benefit from medically induced preterm birth and which will not. Overall, we see infant and fetal mortality rates going down, even as preterm birth rates rise.^39^ A 2004 report from the National Center for Health Statistics gives a better picture of how widespread the improvements have been. They note improvements not just in the infant mortality rate (death before 1 year of age) but in the neonatal mortality rate (death before 28 days of age) and the late fetal mortality rate (death, *in-utero*, after 20 weeks of gestation). They summarize these gains:

Over the more recent period, 1990 to 2001, the IMR (infant mortality rate) declined 26 percent (from 9.2 to 6.8 per 1,000) for an average decrease of 3 percent per year. Between 1990 and 2001 the neonatal mortality rate declined from 5.8 to 4.5 per 1,000 (down 22 percent). Between 1990 and 2001, the late fetal mortality rate declined fairly steadily, by 23 percent, from 4.3 to 3.3 per 1,000. Although the pace of decline has slowed somewhat since the mid-1990s, significant declines in late fetal mortality and infant mortality have been observed through 2001 despite substantial increases in preterm and low birth weight risk, two important predictors of perinatal health.^40^

These paradoxical results suggest that our way of thinking about the associations between prenatal care, preterm birth, and infant mortality may no longer accurately reflect epidemiological, medical, or social realities. Lower preterm birth rates may no longer be the best measure of the efficacy of prenatal and perinatal care. Instead, the best measure may be a combination of the rates of preterm birth, infant mortality, and fetal death.

What are the implications of this analysis for predicting future trends in preterm birth rates? These multiple factors do not allow an easy answer to the question of the optimum mix of antenatal monitoring, interventionist obstetrics, and traditional midwifery approaches to achieve the best possible outcomes. Overall, though, it seems clear that the goals set out by public health authorities in the 1980s and 1990s—for preterm birth rates of 5% and C-section rates of 15%—are probably not optimum. Given current scientific knowledge, they would only be achievable at the cost of rising rates of fetal death or infant mortality. But will the rate of preterm birth continue to rise? That, too, seems unlikely. The recent drops in preterm birth rates in the United States may reflect a new equilibrium, in which advances in prenatal diagnosis, obstetric care of high-risk pregnancies, and neonatal intensive care, along with a new steady state in the demographics of childbearing, and more careful use of assisted reproductive technologies all combine to lead to an optimum balance between reproductive freedom, obstetrical intervention, and perinatal outcomes.
